# Prevalence of and risk factors for severe malaria caused by *Plasmodium* and dengue virus co-infection: a systematic review and meta-analysis

**DOI:** 10.1186/s40249-020-00741-z

**Published:** 2020-09-22

**Authors:** Manas Kotepui, Kwuntida Uthaisar Kotepui, Giovanni De Jesus Milanez, Frederick Ramirez Masangkay

**Affiliations:** 1 Medical Technology, School of Allied Health Sciences, Walailak University, Tha Sala, Nakhon Si Thammarat, Thailand; 2 Department of Medical Technology, Institute of Arts and Sciences, Far Eastern University-Manila, Manila, Philippines

**Keywords:** *Plasmodium*, Malaria, Dengue, Severe complications, Severity

## Abstract

**Background:**

Co-infection with both *Plasmodium* and dengue virus (DENV) infectious species could have serious and fatal outcomes if left undiagnosed and without timely treatment. The present study aimed to determine the pooled prevalence estimate of severe malaria among patients with co-infection, the risk of severe diseases due to co-infection, and to describe the complications of severe malaria and severe dengue among patients with co-infection.

**Methods:**

Relevant studies published between databases between 12 September 1970 and 22 May 2020 were identified and retrieved through a search of the ISI Web of Science, Scopus, and MEDLINE. The pooled prevalence and 95% confidence interval (*CI*) of severe malaria among patients with *Plasmodium* and DENV co-infection was estimated with a random-effects model to take into account the between-study heterogeneity of the included studies. The risks of severe malaria and severe diseases due to co-infection were estimated with the pooled odds ratio (*OR*) and 95% *CI* with a random-effects model.

**Results:**

Of the 5653 articles screened, 13 studies were included in the systematic review and meta-analysis. The results demonstrated that the pooled prevalence estimate of severe malaria among patients with co-infection was 32% (95% *CI*: 18–47%, *I*^2^ = 92.3%). Patients with co-infection had a higher risk of severe diseases than those with DENV mono-infection (odds ratio [*OR*] = 3.94, 95% *CI*: 1.96–7.95, *I*^2^ = 72%). Patients with co-infection had a higher risk of severe dengue than those with DENV mono-infection (*OR* = 1.98, 95% *CI*: 1.08–3.63, *I*^2^ = 69%). The most severe complications found in severe dengue were bleeding (39.6%), jaundice (19.8%), and shock/hypotension (17.9%), while the most severe complications found in severe malaria were severe bleeding/bleeding (47.9%), jaundice (32.2%), and impaired consciousness (7.43%).

**Conclusions:**

The present study found that there was a high prevalence of severe malaria among patients with *Plasmodium* and DENV co-infection. Physicians in endemic areas where these two diseases overlap should recognize that patients with this co-infection can develop either severe malaria or severe dengue with bleeding complications, but a greater risk of developing severe dengue than severe malaria was noted in patients with this co-infection.

**Trial registration:**

The protocol of this study was registered at PROSPERO: CRD42020196792.

## Background

*Plasmodium* and dengue virus (DENV) infection in humans are caused by the bites of infected female mosquitoes, including *Aedes aegypti* and *Aedes albopictus* for DENV transmission and *Anopheles* mosquitoes for *Plasmodium* transmission [1, 2]. Five species of *Plasmodium* can cause malaria in humans, including *P. falciparum*, *P. vivax*, *P. malaria*, *P. ovale*, and *P. knowlesi* [3], whereas four serotypes of DENVs can cause dengue fever (DF), dengue haemorrhagic fever (DHF), and dengue shock syndrome (DSS) [4].

Malaria and dengue are widespread throughout the tropics and subtropics with an estimated 390 million cases of dengue per year [5], and an estimated 228 million cases of malaria occur worldwide [1]. DENV causes a wide spectrum of diseases, ranging from subclinical disease to severe dengue caused by cross-immunity to other serotypes of dengue virus during secondary infection [2]. Severe dengue is classified by the 2009 World Health Organization (WHO) criteria according to levels of severity and includes severe plasma leakage, severe bleeding, and organ failure [6]. *Plasmodium* infection in humans also manifests a wide range of signs and symptoms, ranging from asymptomatic to severe malaria [7]. Severe malaria is classified by the 2015 WHO criteria and includes impaired consciousness, multiple convulsions, prostration, shock, hypoglycaemia, jaundice, pulmonary oedema, bleeding, metabolic acidosis or acute respiratory distress, severe anaemia, and acute renal failure [8].

In endemic areas where malaria and dengue overlap, co-infection with *Plasmodium* and DENV is common where the specific ecosystem, Environmental, and climatic factors support both infections in an individual [9]. Previous studies have reported *P. falciparum* or *P. vivax* and DENV co-infection in different countries [10–15]. Recently, the first case of severe *P. knowlesi* malaria and DENV co-infection in Sabah, Malaysia, was also reported [16]. It is often difficult to differentiate between the two diseases, as they share similar clinical features and laboratory findings, including fever with other non-specific symptoms [17]. Although the clinical signs and symptoms of malaria and dengue were similar, our previous study demonstrated that neutrophil count, lymphocyte count, mean corpuscular haemoglobin concentration (MCHC), and sex can discriminate between dengue and malaria [18].

An understanding of *Plasmodium* and DENV co-infection is crucial for clinicians in the design and management of treatment strategies for severe disease and its complications. Our previous systematic review and meta-analysis demonstrated that malaria parasitaemia, aspartate aminotransferase (AST) and alanine aminotransferase (ALT) levels in DENV and *Plasmodium* co-infection were lower than those in *Plasmodium* mono-infection, while the platelet count and haemoglobin levels were significantly higher in patients with co-infections [19]. Since both DENV and *Plasmodium* infections can lead to life-threatening complications, they need to be diagnosed early and accurately to permit timely management. Currently, a limited number of meta-analytic studies report on the impact of co-infection on disease severity. An understanding of the prevalence of severe complications among patients with co-infections is necessary for clinicians in designing and managing treatment strategies. Therefore, the primary objective of the present study was to determine the pooled prevalence estimate of severe malaria among patients with co-infection. The secondary objective was to determine the risk of severe malaria, severe dengue, and severe diseases (either severe malaria or severe dengue) caused by *Plasmodium* and DENV co-infection. The third objective was to describe the complications of severe malaria and severe dengue among patients with co-infection. The tertiary objective was to describe the complications of severe malaria and severe dengue among patients with co-infection.

## Methods

### Search strategy

Relevant studies published between 12 September 1970 and 22 May 2020 were identified and retrieved through a search of the ISI Web of Science, Scopus, and MEDLINE databases. The search terms “(*Plasmodium* OR malaria) AND dengue AND (severe OR complicated OR complication)” were used to retrieve the related studies. The details of the search and search terms are provided in S1 Table. If a small number of eligible studies were encountered during the study’s selection, case series reporting severe complications in patients with co-infection were also considered. In addition, additional searches through Google Scholar were performed to maximize the number of included studies and to empower the statistical analysis of the present meta-analysis.

### Definition of severe malaria

Severe malaria was defined according to the 2015 WHO criteria as one or more complications in the presence of *Plasmodium* asexual parasitaemia, including impaired consciousness, multiple convulsions, prostration, shock, hypoglycaemia, jaundice, pulmonary oedema, bleeding, metabolic acidosis or acute respiratory distress, severe anaemia, and acute renal failure [8].

### Definition of severe dengue

Severe dengue was defined according to the 2009 WHO criteria as patients with any of the following features: severe plasma leakage with shock and/or fluid accumulation with respiratory distress, severe bleeding, or severe organ impairment [6].

### Definition of severe diseases

Severe disease was defined according to the WHO criteria for both diseases as patients with either severe malaria or severe dengue [6, 8].

### Inclusion and exclusion criteria

Eligible studies were primary studies that reported the severe complications of patients who were infected with both *Plasmodium* and DENV. The primary studies included cross-sectional observational studies, prospective observational studies, and retrospective observational studies. Case series reporting severe complications in patients with co-infection were also considered if a small number of eligible studies were encountered. Previous studies included case reports or case series in meta-analyses in cases of a relatively rare or neglected disease [20–22]. For laboratory diagnosis of malaria, studies using at least one of the reference standards, including microscopy or PCR, were eligible. For laboratory diagnosis of dengue, studies using DENV detection, nucleic acid detection, antigen detection, immunoglobulin (Ig) M or IgG rapid test, or virus isolation were eligible. Articles had to be written in the English language, and published between 12 September 1970 and 22 May 2020. Case reports, in vitro studies, in vivo studies, letters, reviews, commentaries, and proceedings were excluded as they have insufficient data for a systematic review and meta-analysis.

### Data extraction

One author (MK) extracted data from the included studies into Excel 2010 (Microsoft Corp, New York, USA), which were cross-checked by the second author (KUK). Information on authors, years of publication, study areas, years of the survey, study designs, age range, sex, participants, and the number of individuals with severe isolated dengue, severe isolated malaria, severe malaria due to *Plasmodium* and DENV co-infection, severe dengue due to *Plasmodium* and DENV co-infection, and severe diseases (severe malaria or severe dengue according to WHO criteria) were extracted. The extracted data were transferred from the Excel datasheet to Stata version 14.0 (StataCorp, Texas, USA) and Review Manager version 5.3 (Cochrane Collaboration, UK) for statistical analysis.

### Quality of the included studies

Two authors (MK, KUK) assessed the methodological quality of the included studies independently using the Newcastle-Ottawa Scale (NOS) for assessing the quality of nonrandomized studies in meta-analyses [23]; the NOS assesses the risk of bias concerning three domains (the selection of the study groups, the comparability of the groups, and the ascertainment of exposure). A ‘star system’ rating was used to judge the quality of the included studies based on those three domains. A study was rated as “high quality” if it received seven stars or more out of nine total stars. Disagreements between two authors were resolved by discussion for consensus or by consultation with the third (GDM) or fourth author (FRM).

### Statistical analysis

For the primary outcome of the present study, the pooled prevalence and 95% confidence interval (*CI*) of severe malaria among patients with *Plasmodium* and DENV co-infection were estimated with the “metaprop” command in Stata, with a random-effects model to take into account between-study heterogeneity among the included studies [24]. For the secondary outcome, the risks of severe malaria and severe diseases due to co-infection were estimated with the pooled odds ratio (*OR*) and 95% *CI* by a random-effects model, as the studies had considerable amounts of heterogeneity. The results of the meta-analysis were plotted and demonstrated in forest plots, which were generated by Review Manager version 5.3 (Cochrane Collaboration, UK). The heterogeneity of the included studies was calculated with Cochran’s Q test and *I*^2^ statistics. The heterogeneity of the included studies was noted if the *I*^2^ was greater than 50%. The complications of severe malaria and severe dengue among patients with co-infection were demonstrated as frequencies and percentages.

### Publication bias

The publication bias among the included studies was assessed through the visualization of the symmetry of the funnel plot generated by Review Manager. Symmetry in the funnel plot means no publication bias was found, while asymmetry in the funnel plot means publication bias was suspected.

## Results

### Search results

**Fig. 1:**
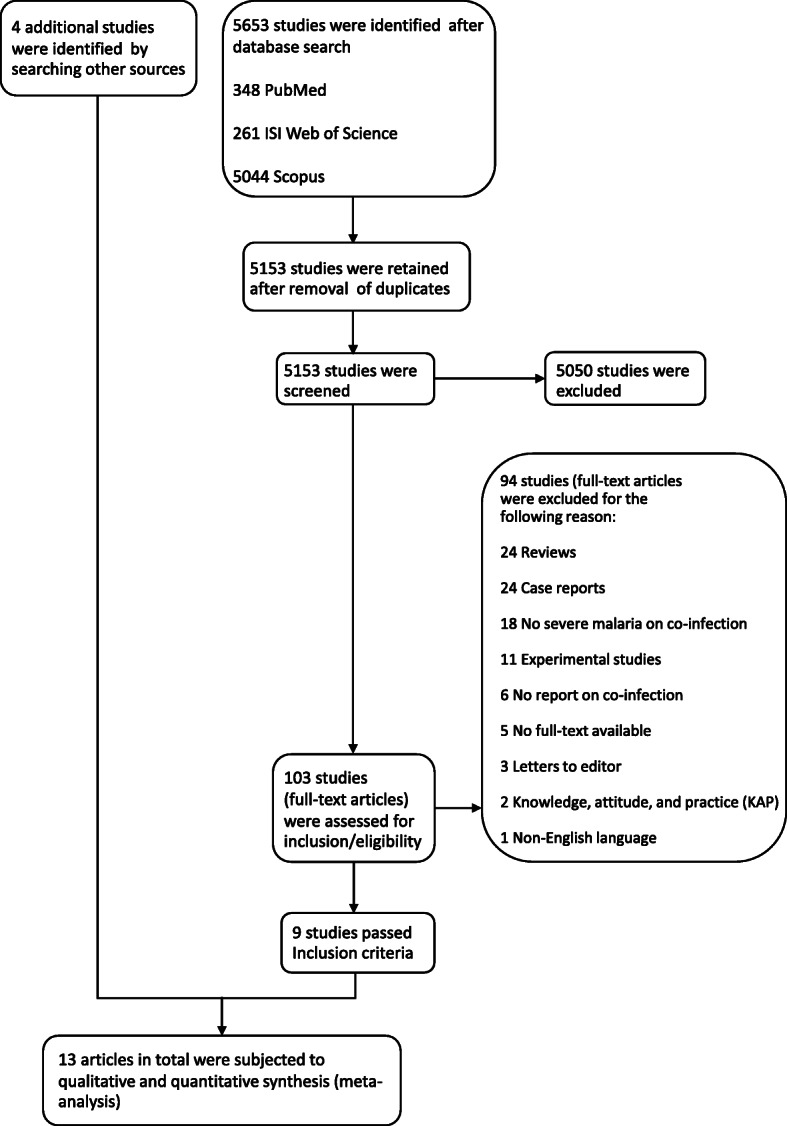
Flowchart for the study selection

The searches of the databases identified 5653 articles. After the removal of duplicates, the titles and abstracts of 5153 articles were screened, of which 103 articles were eligible for full-text examination. Of these 103 articles, 94 were excluded for the following reasons (Fig. 1): 24 were reviews, 24 were case reports, 18 did not evaluate severe malaria in co-infections, 11 were experimental studies, 6 did not report on co-infection, 5 did not have a full text available, 3 were letters to the editor, 2 were knowledge, attitude and practice surveys, and 1 study was not written in English. Overall, 9 studies met the eligibility criteria [11, 13–15, 25–29]. Additional searches through Google scholar retrieved four additional studies [30–33], resulting in 13 studies included in the present study.

### Characteristics of the included studies

The characteristics of the 13 included studies are listed in Table 1. All studies were published between 2009 and 2019 and covered 5 countries. Most of the included studies were conducted in India (7/13, 53.8%), Brazil (2/13, 15.4%), French Guiana (2/13, 15.4%), Pakistan (1/13, 7.7%), and Peru (1/13, 7.7%). Most of the included studies were cross-sectional observational studies (6/13, 46.2%), prospective observational studies (3/13, 23.1%), retrospective observational studies (3/13, 23.1%), and case series (1/13, 7.7%). Most of the included studies performed their studies in adults (6/13, 46.2%), while only one study (1/13, 7.7%) performed a study in children aged less than 15 years. All of the included studies enrolled patients with febrile illness as their participants. Nine studies reported the number of patients with severe disease among patients with co-infection, *Plasmodium* mono-infection, and DENV mono-infection while four studies reported incomplete data on the number of patients with severe diseases. Not enough studies were identified to perform subgroup analyses of the different *Plasmodium* species to determine whether different *Plasmodium* species could result in different disease severities in patients with co-infection.

**Table 1:** Characteristics of the included studies

No.	Author, year, reference number	Study area (years of the survey)	Study design	Age range (mean or median (SD)	Sex (***n***)	Participants	***Plasmodium*** species in co-infection (***n***)	Severe isolated dengue (***n***/***N***)**	Severe isolated malaria (***n***/***N***) **	Severe malaria from dengue virus–***Plasmodium*** co-infection (***n***/***N***) ** ^**a**^	Severe dengue from dengue virus–***Plasmodium*** co-infection (***n***/***N***) ** ^**b**^	Severe diseases (***n***/***N***) **
1.	Ahmad et al., 2016, [25]	India (2012–2013)	Retrospective observational study	Co-infection: 38.6 (16) Malaria: 35.7 (14.5) Dengue: 36.9 (14.9)	Co-infection: male (8), female (1) Malaria: male (35), female (26) Dengue: male (38), female (20)	298 patients with febrile illness	*P. vivax* (9)	11/58 Metabolic acidosis (2), shock (2), seizures (4), death (3)	8/61 Renal failure (2), shock (2), seizures (2), death (2)	1/9 Renal failure (1)	1/9 Renal failure (1), *hypotension (1)	2/9
2.	Ahmad et al., 2019, [30]	India (2017)	Cross-sectional observational study	Co-infection: 7.04 (5.01) Malaria: 5.67 (4.44) Dengue: 10.6 (2.82)	Co-infection: male (10), female (3) Malaria: male (30), female (22) Dengue: male (47), female (27)	164 children with febrile illness	*P. falciparum* (11), *P. vivax* (2)	9/74 Altered mental status (1), haematemesis or melaena (8), death (5), *hypotension (17)	19/52 Altered mental status (8), seizures (4), Jaundice (3), Hypotension (4), death (3)	4/13 Altered mental status (2), seizures (1), hypotension (1), death (1)	5/13 Altered mental status (2), seizures (1), haematemesis or melaena (2), death (1), *hypotension (1)	6/13
3.	Assir et al., 2014, [26]	Pakistan (2012)	Cross-sectional observational study	22 years (range: 12–90 years)	Male (64), female (21)	856 suspected cases of dengue fever	*P. falciparum* (3), *P. vivax* (14)	2/5 Jaundice (2), *hypotension (0)	10/18 Impaired consciousness (1), hypotension (1), bleeding (3), renal failure (1), jaundice (4)	7/17 Hypotension (2), bleeding (5)	5/17 WHO grade 2 bleeding (5), *hypotension (2)	7/17
4.	Barua A and Gill N, 2016, [27]	India (2014)	Cross-sectional observational study	(> 12 years)	NA	156 acute febrile adult cases	NA	2/85 Altered consciousness (1), bleeding (1), *hypotension (4)	15/55 Altered consciousness (2), hypotension (4), bleeding (2), acute renal failure (2), jaundice (2), death (3)	6/16 Hypotension (1), bleeding (2), acute renal failure (1), jaundice (1), death (1)	5/16 Bleeding (2), acute renal failure (1), jaundice (1), death (1), *hypotension (1)	6/16
5.	Carme et al., 2009, [28]	French Guiana (2004–2005)	Retrospective observational study	NA	NA	1723 suspected cases of dengue fever or malaria	*P. falciparum* (3), *P. vivax* (14)	NA	NA	1/17 (*P. vivax*) Severe anaemia (1)	0/17	1/7
6.	Epelboin et al., 2012, [11]	French Guiana (2004–2010)	Retrospective matched-pair study	Co-infection: < 15 (11), > 15 (93) Mean 33.8 years (range: 6 months to 83 years)	Co-infection (104): male (75), female (29)	Patients with malaria and dengue	*P. falciparum* (NA), *P. vivax* (NA)	51/208 Shock signs (5), neurological disorders (8), jaundice (6), haemorrhagic signs (32), *hypotension (3)	39/208* Haemorrhagic signs (25), shock signs (8), neurological disorders (5)	34/104* Haemorrhagic signs (12), shock signs (7), neurological disorders (7)	35/104 Shock signs (7), neurological disorders (7), jaundice (9), haemorrhagic signs (12), *hypotension (10)	34/104
7.	Halsey et al., 2016, [29]	Peru (2002–2011)	Cross-sectional observational study	Co-infection: 29.0 (17.3) Malaria: 29.6 (16.1) Dengue: 27.8 (13.1)	Co-infection: male (12), female (5) Malaria: male (29), female (15) Dengue: male (36), female (15)	Patients with febrile illness	*P. falciparum* (3), *P. vivax* (14)	0/51	3/41 Prostration 3/41	2/15 Prostration 2/15	0/51	2/15
8.	Kamath et al., 2019, [31]	India (2017–2019)	Prospective observational study			778 patients with febrile illness	NA	232/420 Bleeding (178/420), renal failure (54/420), *hypotension (70)	NA	15/15 Bleeding (10), renal failure (1), hypotension (4), death from respiratory distress (1)	12/15 Bleeding (10), renal failure (1), death from respiratory distress (1), *hypotension (4)	15/15
9.	Magalhaes et al., 2012, [13]	Brazil (2009–2010)	Case series	Median 38 (16–97)	Co-infection: male (0), female (11)	132 patients with severe *P. vivax* malaria	*P. vivax* (11)	NA/132	NA	8/11* Jaundice (8), severe anaemia (1), hypotension (1), pulmonary oedema (1)	10/11* Mucosal bleeding (6), *hypotension (1)	10/11
10.	Magalhaes et al., 2014, [14]	Brazil (2009–2011)	Cross-sectional observational study	NA	NA	1578 patients with febrile illness	*P. vivax* (44)	211/584* Jaundice (3), deep bleeding (76)	45/176*	7/44*	32/44* Jaundice (29), deep bleeding (15)	39/44
11.	Mohapatra et al., 2012, [15]	India (2011)	Prospective observational study	NA	Co-infection: male (8), female (29) Malaria: male (68), female (34) Dengue: male (220), female (120)	546 suspected cases of dengue and malaria	*P. falciparum* (24), *P. vivax* (2), mixed infection (1)	140/340 Bleeding 140	90/102* Death (11)	1/27*	10/27 Bleeding 10	11/27
12.	Sonkar et al., 2019, [32]	India (2018)	Cross-sectional observational study		NA	994 patients with febrile illness	*P. falciparum* (16), *P. vivax* (13), mixed infection (1)	NA/279	NA/685	17/30 Jaundice (12), bleeding (5)	NA	17/30
13.	Verma et al., 2016, [33]	India (2015)	Prospective observational study	Adult (> 18 yrs)	Male (256), female (84)	340 patients with febrile illness	NA	38/156 CNS involvement (2), bleeding (32), respiratory distress (4) *hypotension (28)	63/132 CNS involvement (12), bleeding (4), hypotension (4), respiratory distress (20), severe anaemia (20), death (3)	27/52 Bleeding (8), hypotension (3), respiratory distress (4), severe anaemia (12)	12/52 Bleeding (8), respiratory distress (4), *hypotension (3)	26/52
										impaired consciousness (9), multiple convulsions (1), prostration (2), shock/hypotension (19), hypoglycaemia (0), jaundice (21), pulmonary oedema (1), bleeding (42), metabolic acidosis or acute respiratory distress (4), severe anaemia (14), and acute renal failure (3)	Shock (7), respiratory distress (4), severe bleeding (58), renal failure (3), impaired consciousness (9), multiple convulsions (1), jaundice (39)	

* Severe cases defined by authors

** ***n*/*N***: Out of *N* confirmed cases, there are a total of *n* severe cases; NA: not applicable

^a^ WHO malaria- Severity Criteria for Malaria from World Health Organization, 2014

^b^ WHO dengue- Severity Criteria for Dengue from World Health Organization, 2009

### Quality of the included studies

The risk of bias in an individual study was assessed on the basis of the selection of the study groups, the comparability of the groups, and the ascertainment of exposure with a star system rating. Nine studies included in the meta-analysis were rated as “high quality” as they provided data for the primary and secondary outcomes of the present study [11, 14, 15, 25–27, 29, 30, 33]. The other four studies [13, 28, 31, 32] were rated as “adequate quality” as they provided data for one outcome (Table 2).

**Table 2:** Quality of the included studies

No.	Author, year, reference number	Selection				Compatibility	Exposure			Total score (9)	Rating (High, moderate, low quality)
		Is the case definition adequate?	Representativeness of the cases	Selection of controls	Definition of controls		Ascertainment of exposure	Same method of ascertainment for cases and controls	Non-response rate		
1.	Ahmad et al., 2016, [25]	✵	✵			✵✵	✵	✵	✵	7	High
2	Ahmad et al., 2019, [30]	✵	✵			✵✵	✵	✵	✵	7	High
3.	Assir et al., 2014, [26]	✵	✵			✵✵	✵	✵	✵	7	High
4.	Barua and Gill, 2016, [27]	✵	✵			✵✵	✵	✵	✵	7	High
5.	Carme et al., 2009, [28]	✵	✵			✵	✵	✵	✵	6	Adequate
6.	Epelboin et al., 2012, [11]	✵	✵			✵✵	✵	✵	✵	7	High
7.	Halsey et al., 2016, [29]	✵	✵			✵✵	✵	✵	✵	7	High
8.	Kamath et al., 2019, [31]	✵	✵			✵	✵	✵	✵	6	Adequate
9.	Magalhaes et al., 2012, [13]	✵	✵			✵	✵	✵	✵	6	Adequate
10.	Magalhaes et al., 2014, [14]	✵	✵			✵✵	✵	✵	✵	7	High
11.	Mohapatra et al., 2012, [15]	✵	✵			✵✵	✵	✵	✵	7	High
12.	Sonkar et al., 2019, [32]	✵	✵			✵	✵	✵	✵	6	Adequate
13.	Verma et al., 2016, [33]	✵	✵			✵✵	✵	✵	✵	7	High

✵ A star rating

### Prevalence of severe malaria among patients with co-infection

**Fig. 2:**
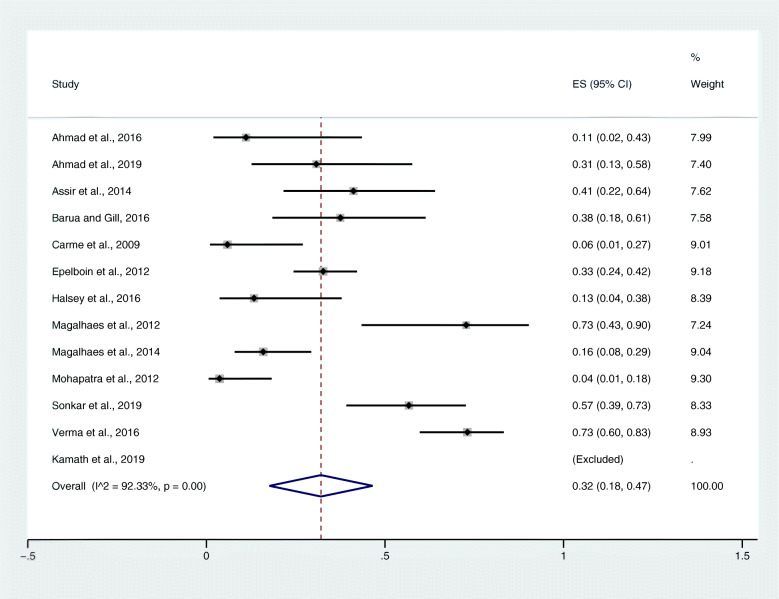
The pooled prevalence estimate of severe malaria/severe complications due to co-infections

From the meta-analysis of the pooled prevalence, there was statistical heterogeneity (*I*^2^ = 92.3%) among the included studies; therefore, a random-effects model was used to produce the pooled prevalence of severe malaria among patients with co-infection. Overall, the pooled prevalence of severe complications/severe malaria of the included studies was 32% (95% *CI*: 0.18–0.47) (Fig. 2). The highest estimated prevalence was found in a study by Magalhaes et al., 2012 (73, 95% *CI*: 0.43–0.90) [13], while the lowest estimated prevalence was found in a study by Mohapatra et al., 2012 (4, 95% *CI*: 1–18%) [15].

### Risk of severe disease among patients with co-infection

**Fig. 3:**
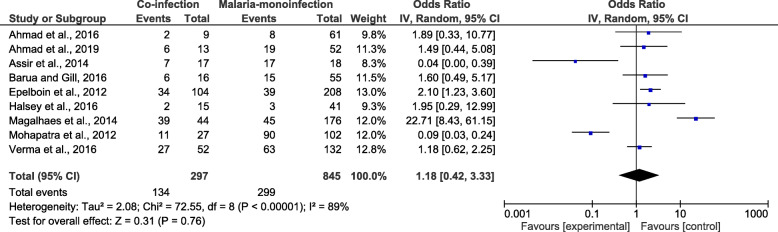
The proportion of severe diseases due to co-infections and *Plasmodium* mono-infections

**Fig. 4:**
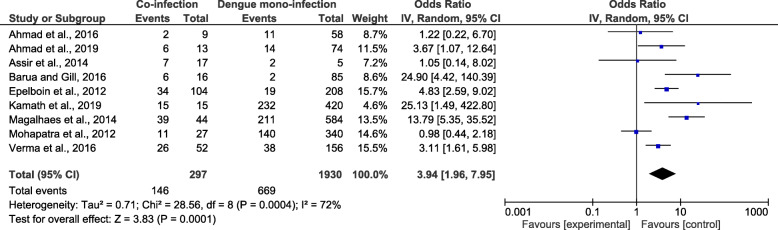
The proportion of severe diseases due to co-infections and DENV mono-infections

As four studies by Carme et al., 2009 [28], Kamath et al., 2019 [31], Magalhaes et al., 2012 [13], and Sonkar et al., 2019 [32] did not report the number of mono-infected patients who developed severe isolated malaria. The meta-analysis of the nine studies demonstrated that patients with co-infection (134/297) and those with *Plasmodium* mono-infection (296/845) had an equal risk of developing a severe disease (*OR* = 1.18, 95% *CI*: 0.42–3.33, *I*^2^ = 89%) (Fig. 3). Nine studies [11, 14, 15, 25–27, 30, 31, 33] were included in the meta-analysis comparing severe diseases due to co-infection and due to DENV mono-infection. The meta-analysis of nine studies demonstrated that patients with co-infection (146/297) and those with DENV mono-infection (669/1930) had a greater risk of developing a severe disease (*OR* = 3.94, 95% *CI*: 1.96–7.95, *I*^2^ = 72%) (Fig. 4).

### Risk of severe malaria among patients with co-infection

**Fig. 5:**
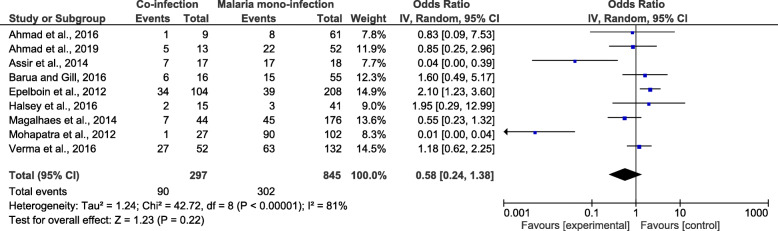
The proportion of severe malaria due to co-infections and *Plasmodium* mono-infections

The meta-analysis of the nine studies included in the meta-analysis demonstrated that patients with co-infection (90/297) and those with *Plasmodium* mono-infection (302/845) had an equal risk of developing severe malaria (*OR*: 0.58, 95% *CI*: 0.24–1.37) (Fig. 5).

### Risk of severe dengue among patients with co-infection

**Fig. 6:**
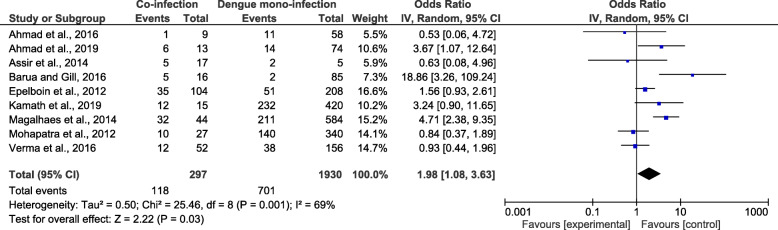
The proportion of severe dengue due to co-infections and DENV mono-infections

The meta-analysis of the nine studies demonstrated that patients with co-infection (118/297) and those with *Plasmodium* mono-infection (701/1930) had a greater risk of developing severe dengue (*OR* = 1.98, 95% *CI*: 1.08–3.63, *I*^2^ = 69%) (Fig. 6).

**Fig. 7:**
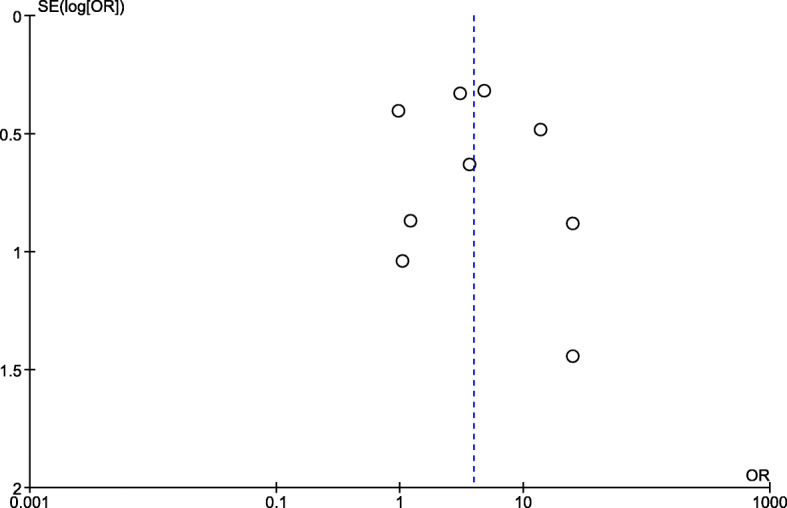
The publication bias among the included studies as demonstrated by funnel plot analysis

### Complications of severe malaria among patients with co-infection

According to the WHO 2015 criteria for severe malaria, most of the severe complications of patients with severe malaria were bleeding (42/106, 39.6%), jaundice (21/106, 19.8%), shock/hypotension (19/106, 17.9%), severe anaemia (14/106, 13.2%), impaired consciousness (9/106, 8.5%), metabolic acidosis or acute respiratory distress (4/106, 3.77%), acute renal failure (3/106, 2.8%), prostration (2/106, 1.89%), multiple convulsions (1/106, 0.94%), and pulmonary oedema (1/106, 0.94%). No case of hypoglycaemia was found.

### Complications of severe dengue among patients with co-infection

According to the WHO 2009 criteria for severe dengue, most of the severe complications of patients with severe dengue were severe bleeding/bleeding (58/121, 47.9%), jaundice (39/121, 32.2%), impaired consciousness (9/121, 7.43%), shock (7/121, 5.79%), respiratory distress (4/121, 3.3%), renal failure (3/121, 2.48%), and multiple convulsions (1/121, 0.83%).

### Publication bias

The publication bias among the included studies was assessed through the visualization of the symmetry of the funnel plot. The results demonstrated that the funnel 340 plot seemed asymmetrical, indicating some publication 341 bias due to the small study effect among the included 342 studies (Fig. 7).

## Discussion

*Plasmodium* and DENV infection are endemic in most tropical and subtropical countries, which are also popular tourist destinations, and these infections are increasingly encountered in travellers who return from areas where malaria is endemic, such as Southeast Asia, Latin America or the Caribbean [34–36]. Those travellers also have a risk of malaria, as the two pathogens share similar geographical areas [37, 38], and the clinical diagnosis and distinction of malaria and dengue is difficult due to their overlapping symptoms [12]. The present systematic review and meta-analysis demonstrated the high prevalence of severe malaria among patients with *Plasmodium* and DENV co-infection (32%). This information indicated that co-infection with *Plasmodium* and DENV impacted the severity of disease in patients with these co-infections. The meta-analysis of severe malaria between patients with co-infection and those with *Plasmodium* mono-infection demonstrated an equal risk of severe malaria between the two groups. This finding might be because most of the included studies were conducted in an adult population, while only one study was conducted in a paediatric population [30]. As most severe malaria or malaria deaths worldwide occur mostly in children aged less than 5 years [1], this result needs to be confirmed by further longitudinal studies. In addition, the comparable risk or severity of disease in patients with co-infection might be because not all of the included studies reported *P. falciparum* and DENV co-infection with *P. falciparum* as the main cause of severe malaria [1]. Although the risk of severe malaria between patients with co-infection and *Plasmodium* mono-infection was comparable, the impact of co-infection on the severity of malaria was reported individually in studies by Epelboin et al., 2012 [11] and Magalhaes et al., 2014 [14], as a significantly higher proportion of severe malaria was found in the co-infected group than in the *Plasmodium* mono-infected group.

The present study demonstrated a significantly higher risk of severe dengue in patients with co-infection than in those with DENV mono-infection. These results indicated that the severe manifestations of co-infection resembled those of dengue. The previous study has supported the results of the present study that the clinical features of dengue are predominant over those of malaria in patients with co-infection [15]. A previous study explained that patients with co-infection presented an immune marker profile resembling that of DENV mono-infected patients [29]. The impact of co-infection on the severity of malaria was reported individually in studies by Ahmad et al., 2019 [30], Carme and Gill 2016 [27], Epelboin et al., 2012 [11], Kamath et al., 2019 [31], Magalhaes et al., 2014 [14], and Mohapatra et al., 2012 [15]. Nevertheless, a possible confounder was that these studies occurred during the dengue epidemic reported by the previous study [27], which might impact this observation.

The definitions of severe complications of severe malaria and severe dengue were guided by the 2014 WHO criteria [8] and the 2009 WHO criteria [6], respectively. Complications such as bleeding, shock, respiratory distress, and severe organ impairments (renal failure, liver failure) can occur in both severe diseases. Our study demonstrated that bleeding and jaundice were the most common complications found in both the severe malaria group and the severe dengue group. Bleeding manifestations were reported in patients with co-infection in 11 included studies [9–11, 13–15, 26, 27, 31–33], while jaundice manifestations were reported in patients with co-infection by five included studies [11, 13, 14, 27, 32]. A previous study demonstrated that bleeding manifestations are uncommon in patients with malaria, and it is difficult to determine the cause of bleeding, as both diseases can induce thrombocytopenia, which leads to bleeding [15]. For the outcome of patients with co-infection, the included studies suggested that the patients’ outcomes were good, as patients with co-infection sought medical treatment earlier than patients with *Plasmodium* mono-infection [39, 40]. Interestingly, a previous study showed that co-infected patients had lower parasitaemia than patients with *Plasmodium* mono-infection, indicating a good outcome [11]. The present study found that deaths caused by *Plasmodium* and DENV co-infection were reported by Ahmad et al., 2019 [30], Barua and Gill, 2016 [27], and Kamath et al., 2019 [31]. The deaths were caused by multiple organ dysfunction syndrome (MODS), as reported in the included study by Kamath et al., 2019 [31].

The present meta-analysis had several limitations. First, the numbers of patients with severe diseases caused by *Plasmodium* and DENV co-infection or mono-infection were reported in four studies, while the other included studies reported the numbers of patients with severe complications of each disease separately. As one patient can develop more than one complication during hospitalization, the results of the pooled prevalence estimates of severe malaria tend to be higher than usual and, therefore, need to be carefully interpreted by readers. Second, hypotension was not included in the comparison of severe diseases between co-infection and mono-infection. Although hypotension is not a 2009 WHO criterion for severe dengue, it is an early sign of shock and is used to determine whether patients need to be treated accordingly. Third, a limited number of studies reported the severe manifestations of these two diseases in the research databases, which caused the low number of included studies in the present meta-analysis.

The manifestations of these two diseases are clinically indistinguishable. In addition, the diagnosis of one disease in a febrile patient in an endemic area does not preclude infection with the other parasite. Therefore, the possibility of *Plasmodium* and DENV co-infection should be confirmed before the final diagnosis. Further well-designed prospective studies are needed to understand the effect of co-infection on the severity of the disease.

## Conclusions

The present study found that there was a high prevalence of severe malaria among patients with *Plasmodium* and DENV co-infection. Physicians in endemic areas where these two diseases overlap should recognize that patients with this co-infection can develop either severe malaria or severe dengue with bleeding complications, but a greater risk of developing severe dengue than severe malaria was noted in patients with this co-infection.

## Supplementary information

**Additional file 1: Table S1.** Search terms.

## Data Availability

All data are available in the manuscript and supplementary files.

## Abbreviations

DENV: Dengue virus
*OR*s: Odds ratio
*CI*: Confidence interval
WHO: World health organization
MCHC: Mean corpuscular haemoglobin concentration.
AST: Aspartate aminotransferase
ALT: Alanine aminotransferase
Ig: Immunoglobulin
NOS: Newcastle-Ottawa scale
