# Supplementary material for: Prevalence of and risk factors for severe malaria caused by Plasmodium and dengue virus co-infection: a systematic review and meta-analysis
Source: Infect Dis Poverty. 2020 Sep 22;9:134. doi: 10.1186/s40249-020-00741-z (PMC7510069; doi:10.1186/s40249-020-00741-z)
Supplement: Supplementary file 1 — Additional file 1: Table S1. Search terms. [file 40249_2020_741_MOESM1_ESM.docx]

**Co-infection of *Plasmodium* spp. and dengue virus leading to severe dengue but not severe malaria complications: A systematic review and meta-analysis**

Manas Kotepui^1*^, Kwuntida Uthaisar Kotepui^1^, Giovanni D Milanez^2^, Frederick R Masangkay^2^

^1^Medical Technology, School of Allied Health Sciences, Walailak University, Thasala, Nakhon Si Thammarat, Thailand

^2^Department of Medical Technology, Institute of Arts and Sciences, Far Eastern University-Manila, Manila, Philippines

Authors’ Email Address:

**^*^Corresponding Author**: Manas Kotepui; manas.ko@wu.ac.th, +66954392469

Kwuntida Uthaisar Kotepui; kwuntida.ut@wu.ac.th

Giovanni D Milanez; gmilanez@feu.edu.ph

Frederick R Masangkay; frederick_masangkay2002@yahoo.com

**Table S1 Search term**

| **Databases** | **Search terms** | **Date** |
| --- | --- | --- |
| PubMed | (Plasmodium OR malaria) AND dengue AND (severe OR complicated OR complication) | 22 May 2020 |
| Scopus | (Plasmodium OR malaria) AND dengue AND (severe OR complicated OR complication)  Search option: All fields | 22 May 2020 |
| ISI Web of Science | (Plasmodium OR malaria) AND dengue AND (severe OR complicated OR complication)  Search option: All fields | 22 May 2020 |
